# Transparent CoAP Services to IoT Endpoints through ICN Operator Networks †

**DOI:** 10.3390/s19061339

**Published:** 2019-03-17

**Authors:** Hasan Mahmood Aminul Islam, Dmitrij Lagutin, Antti Ylä-Jääski, Nikos Fotiou, Andrei Gurtov

**Affiliations:** 1 Department of Computer Science, Aalto University, Espoo 02150, Finland; antti.yla-jaaski@aalto.fi; 2 Department of Communications and Networking, Aalto University, Espoo 02150, Finland; dmitrij.lagutin@aalto.fi; 3 Department of Informatics, Athens University of Economics and Business, Athens 11362, Greece; fotiou@aueb.gr; 4 Department of Computer and Information Science, Linköping University, SE-581 83 Linköping, Sweden; andrei.gurtov@liu.se

**Keywords:** CoAP, IoT, ICN

## Abstract

The Constrained Application Protocol (CoAP) is a specialized web transfer protocol which is intended to be used for constrained networks and devices. CoAP and its extensions (e.g., CoAP observe and group communication) provide the potential for developing novel applications in the Internet-of-Things (IoT). However, a full-fledged CoAP-based application may require significant computing capability, power, and storage capacity in IoT devices. To address these challenges, we present the design, implementation, and experimentation with the CoAP handler which provides transparent CoAP services through the ICN core network. In addition, we demonstrate how the CoAP traffic over an ICN network can unleash the full potential of the CoAP, shifting both overhead and complexity from the (constrained) endpoints to the ICN network. The experiments prove that the CoAP Handler helps to decrease the required computation complexity, communication overhead, and state management of the CoAP server.

## 1. Introduction

The Internet-of-Things (IoT) is anticipated to interconnect billions of smart objects and constrained devices that will generate an enormous amount of information. Cisco forecasts that there will be around 50 Billion IoT devices such as radio-frequency identification (RFID) tags, sensors, and actuators on the Internet by 2020 [1]. IoT is increasingly relying on data and information rather than on end-to-end communication, which could promote the adoption of an Information-Centric Networking (ICN) architecture [2]. The driving paradigm in ICN is that Named data is the central element of ICN communication. To achieve this, ICN introduces named content as a network primitive and provides in-network caching. ICN allows the user to retrieve a particular content regardless of any reference to the physical location of the content. However, considering IoT scenarios from an ICN perspective may raise challenges of a very dynamic and heterogeneous nature. First, the information exchanged in IoT environment is heterogeneous. During the last decade, many custom proprietary solutions have been developed and deployed in a multitude of the IoT use cases. However, the lack of interoperability among these solutions exposes a major challenge. Second, many of the IoT devices have limited battery capabilities and, therefore, the networking and computing capabilities of the IoT devices have to be energy efficient for long-life operation. Third, the ICN approach in IoT needs not only refactor the networking protocols and Internet applications [3]. This is because ICN introduces named data networking as a replacement of Internet Protocol (IP). ICN has no notion of host at its lowest level, i.e., ICN integrates content/service/host abstraction, name based routing, and in-networking caching as parts of the network Infrastructure. Such evolutionary changes to every network layer are an overwhelming barrier to the adoption of ICN. With this in mind, the POINT project [4] proposes an evolutionary approach to ICN adoption: an individual ICN operator uses network attachment points (NAPs) in order to translate legacy IP application traffic to ICN, i.e., the ICN network is transparent to IP endpoints.

In this paper, we assume or target an operator network which runs ICN in its network core. Outside the operator network, the conventional Internet based on IP protocol performs communication between user endpoint and the Network Attachment Point (NAP) which is a gateway between ICN and IoT technologies. Our design approach connects IP based Endpoint through ICN. The user endpoint (UE) using legacy IP is oblivious to ICN. We called our solution which enables the legacy CoAP [5] and CoAP extensions to IoT devices through the ICN operator network so that the IP endpoints implementing only the core CoAP can benefit from the CoAP and its extensions by leveraging the multicast capabilities of the POINT architecture. We connected two converging, yet not totally compatible, issues: Information Centric Networking (ICN) and IoT specific communication protocols (CoAP handler) through the Network Attachment Point (NAP). The communication between IP endpoint and NAP is based on IP. Then, NAP performs protocol translation and forwards the data to other IP endpoint. The NAP which is close to a client is called client NAP (cNAP) and server side NAP is called sNAP. In this paper, we cover all CoAP specific communication scenarios through ICN. For this NAP does fine tune engineering in the whole system.

The main goal of this paper is introducing CoAP for the ICN NAP that improves the IP-based CoAP service offering of the clean slate ICN operator network. The key idea of this architecture is that we target a single operator network which would like to improve its own IP-based services. Our approach enables the usage of the legacy CoAP and CoAP extensions, i.e., our system architecture allows for connecting existing CoAP protocols running on IoT devices without any modification. Therefore, the design principle of this architecture allows for providing transparent CoAP services to IoT devices through an ICN core network, while shifting both complexity and communication overhead from the CoAP endpoints to the ICN core network.

**Figure 1:**
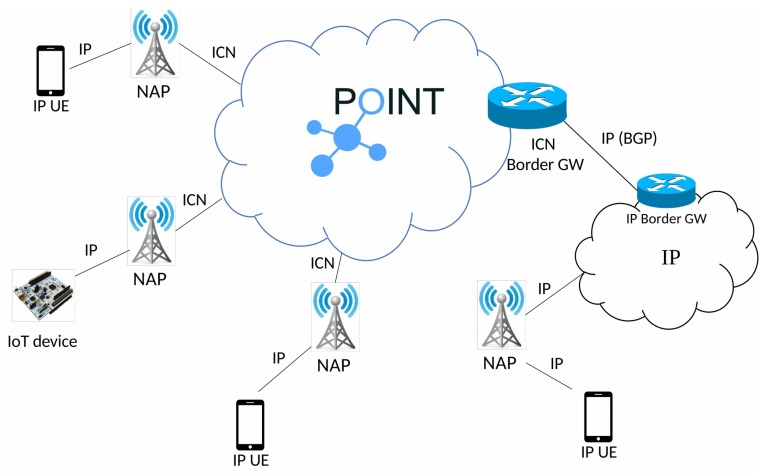
POINT architecture.

Furthermore, in this work, we have not assumed that the two technologies are similar. Nevertheless, both ICN and CoAP has some shared principles in their design (e.g., Pub/Sub based CoAP observe [6]). Section 5.3 highlights the outcome of our design in terms of computation complexity and communication overhead through inherent ICN multicast. In addition, the key benefit is that the CoAP server does not need to implement IP multicast and any modification to the Domain Name System (DNS) is not required. In summary, the design and implementation of the for the POINT architecture (Figure 1) which enable the legacy CoAP and CoAP extension to IoT devices through the ICN operator network. Through our approach, the IP endpoints implementing only the core CoAP can benefit from the CoAP and its extensions by leveraging the multicast capabilities of the POINT architecture.

CoAP protocol has various extensions, such as CoAP observe [6] and group communication [7], that provides the potential for developing novel IoT applications. Despite its potential, a full-fledged CoAP deployment on the resource-constrained devices poses great challenges [8]. For example, in order to support delay-tolerant messaging or publish-subscribe communication (i.e., CoAP observe), CoAP servers should maintain extensive state for CoAP clients and require significantly higher processing capabilities. In addition, CoAP group communication could be implemented by using IP multicast [9], with DNS mapping of group names (included in the CoAP UR requests) onto the appropriate IP multicast address. To support group communication, CoAP servers should implement IP multicast and, for each group, a specific IP multicast address should be assigned. Therefore, the resource-constrained IoT devices need to allocate more resources for a DNS client implementation. In order to tackle these challenges, the inherent features of ICN have a great potential of deploying IP based CoAP in resource-constrained IoT devices. Therefore, the main goal of this paper is to improve the IP-based CoAP service offering of the ICN operator network without any adaptations to the CoAP endpoint. In addition, the CoAP endpoint must be oblivious to ICN.

In this paper, we present the design and implementation of the CoAP handler for the POINT architecture which enable the legacy CoAP and CoAP extension to IoT devices through the ICN operator network so that the IP endpoints implementing only the core CoAP can benefit from the CoAP and its extensions by leveraging the multicast capabilities of the POINT architecture. The summary of the key contributions is as follows:

- Enabling CoAP group communication without IP multicast support and any modification to the DNS.
- Enabling CoAP observe extension support transparently, i.e., CoAP requests for the same IoT resource are aggregated in the NAP, hence only a single CoAP client is visible to the CoAP server.
- Enabling delayed response in the case that the resource is not available or the CoAP server is in the sleep mode.

Our earlier efforts [10,11] present the potential of the ICN architecture to improve the performance of IoT endpoints which run CoAP and illustrates how various CoAP-specific communication scenarios could benefit from an ICN network. Based on the analysis of [10], we present the preliminary design, implementation, and qualitative evaluation (e.g., communication overhead, state management of IoT devices) of the CoAP Observe extension over ICN in [11]. The CoAP observe extension has been implemented in a proxy which is connected to the NAP of the POINT architecture. Moreover, CoAP group communication is demonstrated in [12]. In contrast, this work presents the design, implementation, and experiment with the in the NAP that provides the CoAP services (e.g., CoAP Observe, delayed response, group communication) to IoT devices through the ICN core network. The CoAP and its extensions are implemented in the NAP so that it can support all communication scenarios discussed in [10]. This approach opens the direction of future developments of IoT services through the inherent features of ICN (e.g., request aggregation, ICN multicast).

The remainder of this paper is organized as follows. In Section 2, we introduce the CoAP and CoAP extensions as well as the POINT architecture. In Section 3, we explain the problem statements and how our solution handles those. Section 4 presents the design and implementation of the CoAP Handler for the POINT architecture that provides the transparent CoAP services to the IoT devices through the ICN operator network. Then Section 5 discusses the experiments of our approach. Section 6 presents the related work and, finally, Section 7 concludes our paper.

## 2. Background

### 2.1. CoAP

The Constrained Representational State Transfer (REST)ful Environments (CoRE) working group has designed and developed Constraint Application Protocol (CoAP) [5] which is intended to operate in constrained IP networks and provides RESTful services in constrained devices. The CoAP protocol has been developed as a replacement of HTTP for the IoT. Unlike HTTP, CoAP operates under a simple framework consisting of the following two layers on top of User Datagram Protocol (UDP): the Messaging layer and the Request/Response layer. The messaging layer handles the asynchronous nature of communication and deals with User Datagram Protocol (UDP), while the request/response layer deals with request/response messages using method and response codes. The messaging model supports four types of messages: CON (confirmable), NON (non-confirmable), ACK (Acknowledgement), RST (Reset). The CON message is used to provide a lightweight optional reliability to the CoAP protocol. If a CON request message cannot be answered, the recipient sends RST message instead of ACK. The request/response layer lies on top the messaging layer and deals with request/response message. The CoAP request and response semantics are included in CoAP messages using Method or Response code, respectively. If a CoAP client sends a CON request message to a CoAP server, the response message is generated by the CoAP server with type ACK and sent to the CoAP client immediately. The response data is carried in the ACK, which is called a piggybacked response. If the CoAP sever is not ready to respond immediately, it sends a response message with an empty ACK. If the client receives the empty ACK, it stops retransmitting the same request.

Various CoAP extensions enable novel applications, departing even further from the traditional client/server model. For instance, CoAP observe is described in [6] as an extension to the CoAP protocol. CoAP observe enables a client to observe a resource through a simple publish/subscribe mechanism. CoAP group communication is another CoAP extension defined in RFC 7390 [7]. The underlying mechanism of CoAP group communication is sending a single CoAP message to a specific group of devices by exploiting UDP/IP multicast for the requests and unicast UDP/IP for the responses. A CoAP group is created by a set of CoAP servers, where each server is configured to receive CoAP group communication requests destined to the group’s associated IP multicast address. A CoAP group name to IP multicast address mapping is added to the appropriate DNS servers.

### 2.2. The POINT Architecture

The POINT architecture allows the legacy IP traffic to be run over an ICN core network; the ICN is mainly deployed at a single network operator [4]. The core ICN in the POINT network follows the publish/subscribe principle originally developed in the PSIRP/PURSUIT projects [13]. This architecture divides the core network functions into a control and data plane. At the Control plane, the architecture has Topology manager (TM) which creates a distributed awareness of the local network topology. The TM discovers the network topology by executing a distributed routing protocol, e.g., Open Shortest Path First (OSPF) [14]. On top the topology management, the architecture has Rendezvous (RV) system which takes the responsibility of matching between the publishers and subscribers. Whenever the RV finds a matching between the publisher(s) and subscriber(s), it requests the topology manager to create a logical information delivery tree from the publisher(s) to the subscriber(s). The Data plane finally takes the responsibility of Forwarding (F) functionality to perform the actual data transfer between subscriber and publisher.

The POINT architecture follows a gateway based approach where the first link from the user endpoint (UE) to the network is based on IP-based protocols. The Network Attachment Point (NAP) serves as the entry point into the ICN network as shown in Figure 1. NAP provides a number of handlers for existing IP-based protocols (e.g., HTTP, CoAP, and basic IP) that map the underlying protocols onto appropriate named objects within the ICN core. The communication between user equipment (UE) and NAP follows the standard IP-based protocols. The other communication interfaces between NAP and ICN are publisher-rendezvous (PR), subscriber-rendezvous (SR) and Topology manager-publisher (TP).

Every content item in the POINT architecture is identified by a flat identifier known as the Rendezvous Identifier (RId). Moreover, every content item belongs to at least one scope. The purpose of a scope is to group “similar” content items and to give a hint about content location. Scopes are hierarchically organized and identified by a Scope Identifier (SId). Scopes are managed by specialized Rendezvous Nodes (RNs), which form an overlay Rendezvous Network. The rendezvous network provides a lookup service, which routes a “subscription” to a RN that “knows” (at least) one publisher for the requested item. A typical ICN transaction in POINT involves the following steps. A content item is assigned a RId and stored in (at least) one publisher that advertises its availability in one or more scopes. With this advertisement, the RId is stored in the RNs that manage these scopes. Subscribers send subscriptions for specific (SId, RId) pairs, which are routed by the rendezvous network to an appropriate RN. Upon receiving a subscription message, and provided that at least one publisher exists, the RN instructs a Topology Manager to create a forwarding path from a publisher to the subscriber, which is included in the notification message to the publisher. Finally, the content item is transferred from the publisher to the subscriber.

## 3. Scenario and Problem Statement

### 3.1. Scenario

Let us consider a smart apartment which is referred as SensiHome. SensiHome can include controllable lightning fixtures, infra-red sensors for movement detection, temperature sensors, ambient light sensors, a number of cameras to monitor the entrance and other locations, and controllable window shades. SensiHome includes a local home controller, which binds the functions of the sensors and actuators together to form a functional system. The sensors and actuators use the Constrained Application Protocol (CoAP) to communicate with the local controller and/or other network entities. Let us assume that SensiHome is a partner with a mobile operator called FutureTel. The operator connects all controllers to SensiHome’s network through FutureTel’s network so that SensiHome could allow its customers to control their smart apartments using their mobile phones and a FutureTel subscription. In the following, we discuss the potential challenges of this scenario and what is the potential of our solution in tackling of these challenges.

### 3.2. CoAP Group Communication

SensiHome may periodically prompt many sensors, actuators, and controllers, creating bursts of traffic. CoAP group communication can alleviate this phenomenon. Nevertheless, this requires IP multicast support with DNS mapping group names (included in the CoAP Uniform Resource Identifier (URI) requests) onto the appropriate IP multicast address. With this approach, the CoAP servers should implement IP multicast and, for each group, a specific IP multicast address should be assigned (and configured in the CoAP endpoints). Therefore, there is a need for supporting resolution systems that translate resource URIs into IP addresses. However, it is difficult for constrained devices to allocate more resources for a DNS client implementation.

In our approach, CoAP server does not need to implement IP multicast. In addition, any modification to the DNS is not required. Group names are transparent to CoAP server since the is responsible to manage the group names. Furthermore, there is no necessity of mapping a group name a priori to a lower layer network address. Therefore, the resource-constrained IoT devices do need to allocate more resources for a DNS client implementation and IP multicast.

### 3.3. CoAP Observe

Request aggregation is not common in the conventional IP network. There are some cases where the constraint devices (e.g., temperature sensors) may receive a large number of requests that require significantly higher processing capabilities. In the case of CoAP observe, a CoAP server needs to maintain state for each client and respond separately to each of them.

The details of supporting CoAP observe extension in the NAP of the POINT architecture is presented in [11]. In brief, CoAP requests for the same resource are aggregated in the NAP, hence only a single CoAP client is visible to the CoAP server. In addition, when the status of a resource changes, the updates are transmitted using ICN multicast, thus safeguarding network resources.

## 4. Design and Implementation

In this section, we will discuss the design and implementation of the CoAP Handler in the NAP of the POINT architecture. The design and implementation of the CoAP Handler take into account the various CoAP-specific communication scenarios (e.g., the CoAP observe, group communication) as presented in our earlier CoAP over ICN work [10]. The details of the algorithms supporting CoAP observe in the NAP is presented in [11]. The basic idea of our solution is that the CoAP Handler receives CoAP requests from CoAP clients (over IP), performs protocol translation from CoAP messages to ICN messages and forwards the requests to the CoAP server. The CoAP server generates a response which is forwarded to the CoAP clients following the reverse process. The ICN network is oblivious to the CoAP endpoints.

### 4.1. Network Setup

**Figure 2:**
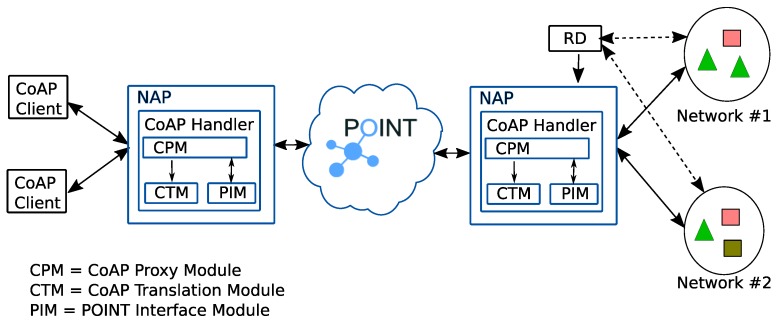
An example of CoAP over ICN reference architecture. On the right there are Things offering resources. Each resource is specified by a color and also by shape. On the left there are the CoAP clients.

Figure 2 illustrates the network setup for our solution. In the middle of the figure, there is the POINT network that interconnects NAPs. In the right part of the figure, there are networks of “Things”. Each Thing acts as a CoAP server offering a resource; the same type of resource can be offered by many Things located in different networks (e.g., there can be many sensors deployed in various parts of a city offering temperature measurements). Each network of Things is connected to the POINT network through a NAP. A network of Things may be directly attached to a NAP. A CoAP Resource Directory (RD) hosts the descriptions of resources provided by the CoAP servers. In the left part of the figure, there are CoAP clients. A CoAP client is also connected to the POINT network through a NAP.

### 4.2. Functional Requirements

The following requirements are necessary and essential considering designing a in the NAP to provide transparent CoAP services to the IoT devices:

- The CoAP protocol runs on top of the UDP. A CoAP Handler MUST maintain the state of the CoAP clients prior to forwarding their requests to the POINT network. This enables the CoAP Handler to forward the corresponding response back to the appropriate client.
- Efficient state maintenance needs to be designed within the CoAP Handler in the case of resource subscription showcasing the ICN benefits to the CoAP observe extension. A CoAP Handler should maintain additional state when similar requests are issued by multiple clients attached to the same client-side NAP (cNAP). Similarly, server-side NAP (sNAP) should also consider how to efficiently handle multiple requests for the similar resource from multiple cNAPs.
- The CoAP Handler MUST follow the protocol semantics of CoAP and its extension in the NAP so that the legacy IP application is oblivious to ICN.

### 4.3. Communication Scenario

The CoAP Handler considers the following communication scenarios as CoAP services to CoAP endpoints:

- Single request and immediate response: The CoAP server is ready to send the response immediately.
- Single request and delayed response: This scenario happens when the server is sleeping for a while and the resource is not yet available.
- CoAP observe extension: The CoAP client is interested in the update notification of a particular resource.
- CoAP group communication: The client is interested in multiple resources hosted on multiple IoT devices.

### 4.4. The Components of the CoAP Handler

A CoAP Handler is composed of the following components Figure 2:

CoAP proxy Module: The proxy component of the CoAP Handler is responsible for IP connectivity between a NAP and CoAP endpoint(s). The communication is based on UDP. The proxy receives a CoAP request from a CoAP client. Upon reception of the CoAP messages, this component extracts the necessary and essential information from the CoAP messages, gives an instruction for a specific action (e.g., single request-single response, CoAP observe, etc.), manages states and allows for translating CoAP messages to ICN messages.

Protocol Translation: This component is a minimal implementation of the CoAP protocol following the semantics of RFC 7252 which is utilized to translate CoAP messages to ICN messages.

POINT Interface Module: This module advertises ICN messages (i.e., translation of CoAP requests) to the ICN network. The rendezvous node of ICN network takes the responsibility of forwarding these CoAP messages to the appropriate NAP(s). Finally, a NAP which receives an ICN message forwards the original CoAP request to the CoAP server. The CoAP server follows the reverse path to forward the response to the CoAP clients.

### 4.5. Namespace

**Figure 3:**
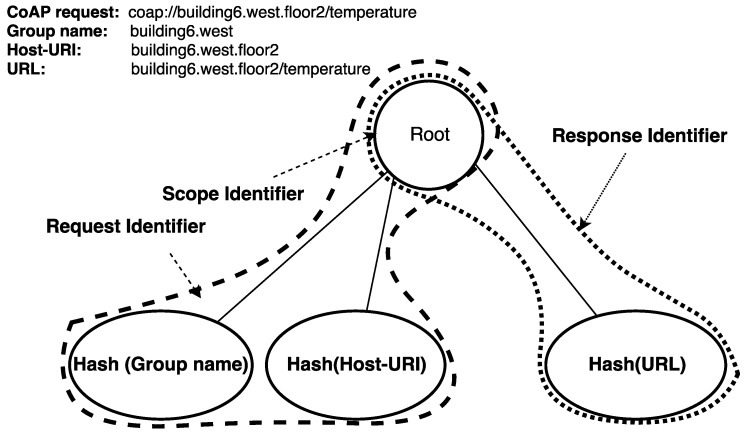
Namespaces for in the POINT architecture.

To realize CoAP communication, the CoAP Handler utilizes the namespace depicted in Figure 3. A CoAP server is identified by a Host-URI and it may be part of multiple CoAP groups. The namespace within the ICN enabled operator network is grouped under a single scope (illustrated with Root). A CoAP request to a CoAP server is a publication to its fully qualified domain name (FQDN) represented by the Request Identifier, which is generated by hashing over Host-URI as shown in Figure 3. In the case of CoAP group communication, a request to a CoAP group is a publication to the hash of the Group-name. A response is represented by Response Identifier which is generated by hashing over URL as shown in Figure 3.

### 4.6. ICN Operations

A request from a CoAP client is translated to an appropriate ICN name which corresponds to the FQDN of the CoAP server, while the response to that request is a publication to the appropriate ICN name corresponded to the URL of the request. This enables a sNAP to subscribe to the FQDN of any attached CoAP server, while a sNAP can publish the CoAP response to the appropriate URL. Let us describe a simple example of a CoAP client request and CoAP server response. The sNAP starts subscribing to the hashed Host-URI of the CoAP server. Then, cNAP receives a CoAP request for the URL “aueb.example.gr” which is published to the Rendezvous (RV). The RV looks up for matching the request with the server’s subscription and requests the TM to create a forwarding path for the request as well as the reverse path. Eventually the TM sends the forwarding path (FID${}_{req}$) and reverse path (FID${}_{res}$) to the cNAP. The cNAP transfers the CoAP message to the sNAP using (FID${}_{req}$). The sNAP decapsulates the CoAP message and sends it to the CoAP server. The CoAP server receives the CoAP message and forwards the CoAP response to the sNAP. The sNAP forwards it to the cNAP using (FID${}_{res}$). Finally, the cNAP forwards the response to the appropriate client.

### 4.7. Request Processing

Algorithms 1 and 2 present the process of handling CoAP requests of different types as discussed in Section 4.3 in a cNAP and sNAP. Upon reception of a CoAP request from a CoAP client, cNAP checks the request packet to verify the request type. cNAP also looks up its state table to verify if there is an entry for this request. If no match is found, cNAP creates an entry for this request and publishes it to the ICN network. Otherwise, the cNAP aggregates the request and appends the requester in that entry of the state table. If there exist multiple resources hosted in different IoT devices which are connected to different sNAPs, ICN multicasts the request to those sNAPs, resulting in group communication. If the request type is CoAP observe, the cNAP follows the algorithm presented in [11]. The sNAP maintains the state of the cNAPs, which request the same resources in a situation, when the resource is not yet available. When the resource is available to the sNAP, it multicasts the response to all cNAPs. This case demonstrates the delayed response communication scenario.

**Algorithm 1** CoAP Request in cNAP

isProxyOption←[coap_request]

coap_header←[coap_request]

Token←[coap_request]

request_type←[coap_request]
**if**request_type = = Confirmable **then** sendemptyACKtorequestingclient
 request_type←Non-confirmable

**end if**
**if**isProxyOption = = true **then** proxyURI←[coap_request]
 split_proxyURI(hostURI,port,resourceURI,query)
 isObserveOption←[coap_request]
 applyrequestaggregation
 request←newcoap_packet()
 request←coap_header
 request←insertOption(hostURI,resourceURI)
 **if** portandqueryarenotNULL **then**  request←insertOption(port)
  request←insertOption(query)
 **end if** **if** isObserveOption = = true **then**  applycoapobservealgorithm
  request←insertObserveOption()
 **end if** **if** coap_requesthaspayload **then**  request←insertPayload()
 **end if** /∗possibilityofICNmulticast
 ∗multipleserverregisterforthisresource
 ∗/
 creatependingICNpublicationListusingToken
 SIDhost←sha256_hash(hostURI)
 SID←COAP_SCOPE_ID+SIDhost
 RID←generateRandomString()
 ICN_ID←SID+RID
 updateICN_ID_to_client_token_list
 $publish\;request\;to\;ICN\;usingICN_{I}D$

**end if**



**Algorithm 2** CoAP Request in sNAP

request_pkt←[ICN_message]

ICN_ID←[ICN_message]

SToken←ICN_ID_To_SToken[ICN_ID]
**if**SToken = = NULL **then** SToken←generateNewToken()
 ICN_ID_To_SToken[ICN_ID]←SToken
 request_pkt←SToken

**else**
 request_pkt←SToken
**end if**

SToken_To_ICN_ID_list[SToken]←ICN_ID

resourceURI←[request_pkt]

coap_server_list←lookup(resourceURI)

/∗groupcommunicationiflist>1∗/

**for** coap_server_list **do**
 coap_server←enque(coap_server_list)
 /∗suppresssimilarrequestfromdifferentcNAP∗/
 **if** pending_res_list(res_ICN_ID,coap_server).size == 1 **then**  sendrequest_pkttocoap_server
 **end if**
**end for**



### 4.8. Response Processing

Algorithms 3 and 4 present the process of handling the CoAP response in a sNAP and cNAP. Upon reception of a CoAP response, sNAP looks up its state table to find a list of cNAP(s) interested in this response. If multiple cNAPs are found, sNAP utilizes the ICN multicast to forward a single copy of this response to those cNAPs. Afterwards, sNAP removes the entry from the state table except the case of the response packet containing the CoAP observe option. Finally, cNAP forwards the response to the appropriate client(s).

**Algorithm 3** CoAP Response in sNAP

token←[coap_response]

ICN_ID←token_To_ICN_ID[token]

response_type←[coap_response]
**if**response_type = = Confirmable **then** sendemptyACKtoserver
 coap_response←setResponseType(Non-confirmable)

**end if**

cNAP_list←pending_response(ICN_ID,server)

//coincidentalmulticastifcNAP_listsize>1

sendcoap_responsetocNAP_list



**Algorithm 4** CoAP Response in cNAP

ICN_ID←[ICN_message]

coap_response←[ICN_message]

token_list←lookup(ICN_ID)

isObserveOption←[coap_response]
**if**isObserveOption = = true **then** observer_list←lookup_observer(token_list)
 sendresponsetoobserver_list

**else**
 client_list←ICN_ID_to_clients_token_list
 sendresponsetoclient_list

**end if**



### 4.9. Implementation

We have implemented CoAP Handler in C/C++. The libcoap library is widely used in IoT. The implementation of the CoAP Handler follows the semantics of RFC 7252 and RFC 7641. Initially, the most challenging task was the CoAP observe extension which has been implemented in the NAP of the POINT architecture. The group communication is implemented exploiting the inherent multicast support of the ICN architecture. Originally, the group communication of the CoAP Handler is based on the software solution, named Blackadder, which has been implemented in the European Union (EU) FP7 project PURSUIT and EU H2020 POINT Project [4].

The POINT architecture allows standard IP traffic to be run over an ICN core network, deployed typically at a single network provider [4]. POINT’s core ICN network is implemented using the Publish-Subscribe Internet (PSI) ICN architecture, a publish-subscribe architecture, where users interested in receiving specific content subscribe to it, while content owners advertise their content and if requested they publish it (i.e., they transfer it to the subscribers by default through multicast).

## 5. Experiment

### 5.1. Experiment Setup

**Figure 4:**
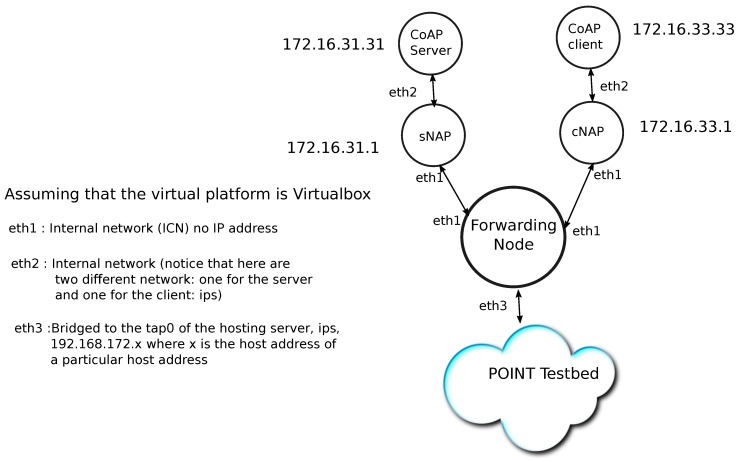
A simple IoT test network.

We have constructed an IoT testbed (Figure 4) which is connected to the existing POINT testbed through a Network Attachment POINT (NAP). The IoT testbed consists of several sites. The generic composition of each site consists of the CoAP Handler of the NAP, an Edge Controller, and the embedded Leaf Nodes. As an example, these form a house automation system controlling lights, heating and cooling systems.The Edge Controller runs a control algorithm and provides user interface. The Leaf nodes includes stm32 nucleo-f401 with 802.3at provided by ELL-i cooperative (https://ell-i.org/). The operating system of the Leaf node is RIOT-OS. The IoT testbed consists of sensors, such as temperature and humidity sensor, luminosity sensor, motion sensor etc., that are attached to the Leaf nodes.

The POINT testbed connects all partners of the POINT project throughout Europe using OpenVPN. The POINT overlay testbed is based on Blackadder ICN platform. We also test our solution in Mininet (http://mininet.org/) environment to demonstrate the applicability of the CoAP Handler in the network.

**Figure 5:**
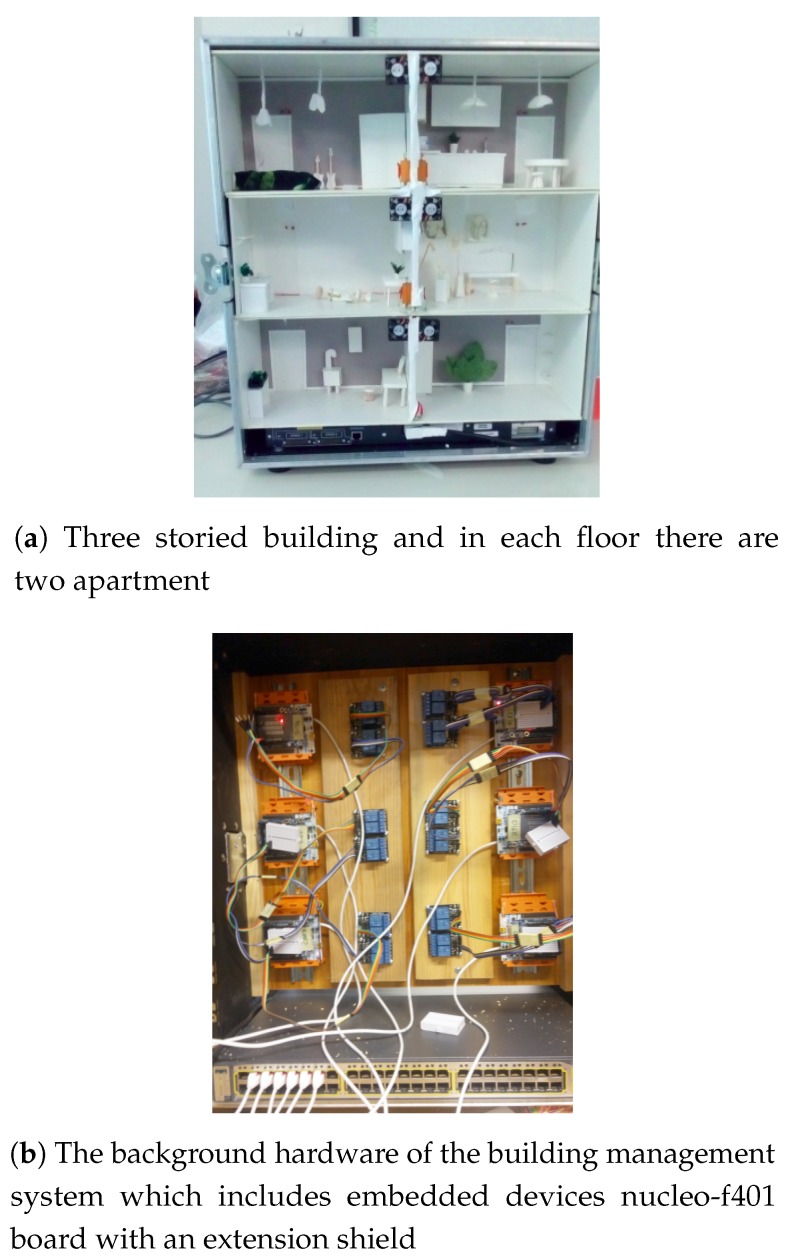
Experiment setup.

**Figure 6:**
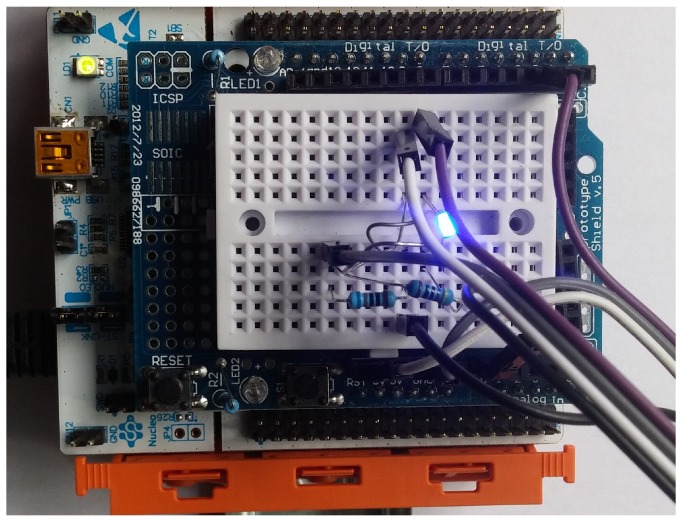
Image of a nucleo-f401 board with an extension shield that is used in our experiment.

In this experiment, we considered a three-floor building with two wings as shown in Figure 5. In our laptop we emulated a POINT network with 6 NAPs (one for each “building area”) using mininet. Each NAP was implemented as a virtual host; the network interface of each such host was bridged with a real network interface so as to be able to transmit data to the real world. Through PoE-enabled switches, each NAP will be connected to a number of nucleo-boards (1 or 2). These boards were using the RIOT operating system and the gcoap library to implement simple CoAP servers that accept requests over IPv6. A CoAP client implemented in our laptop using the Californium CoAP library controls actuators (LEDs and fans) attached to the nucleo boards (Figure 6) and receives observe notifications. The audience is able to interact with the CoAP client through a GUI. The experiment shows that our solution requires no special configuration in the Things’ side. A NAP can easily be configured with a new group name.

The experiment has used the following equipment:

- A laptop that emulates the POINT network using Mininet and POINT’s prototype
- One USB hub where USB Ethernet adapters are attached
- Three Power over Ethernet (PoE) switches, one for each floor of the building
- Ten nucleo-f401 boards with LEDs and fans, powered over Ethernet
- One VLAN per floor.

The implementation details can be found in the following URL: https://github.com/point-h2020/point-3.0.0/tree/master/apps/coap.

### 5.2. Results and Discussion

In this section, we explain how CoAP Handler provides transparent CoAP services to IoT devices through an ICN core network. In addition, we demonstrate how ICN can benefit CoAP and its extensions, shifting both overhead and complexity from the CoAP endpoints to the ICN network.

### 5.3. Group Communication

The POINT leverages its information-centrism and inherent support for multicast to provide seamless group communication among the CoAP endpoints. In particular, it takes advantage of the PSI’s name structure in order to organise group “attributes”; then it assigns values to these attributes to construct group names, and maps these names onto the appropriate PSI scopes. Our system architecture enables a request to be made to a group of CoAP servers that implement the standard version of the CoAP protocol (i.e., they do not need to support RFC 7390). As a result, the CoAP servers do not have to implement IP multicast. Moreover, there is no need for modifications to the DNS. CoAP servers are oblivious to group names, since names are handled by the NAPs. Thus, the ICN core makes group name administration easier: new attributes can be easily added to the namespace without affecting already deployed NAPs. Moreover, group names do not have to be mapped a priori to a lower layer network address.

**Figure 7:**
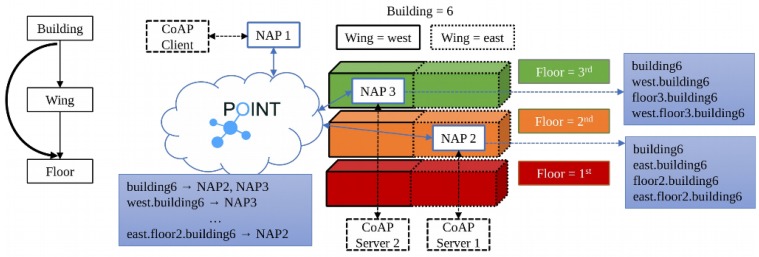
An overview of the group communication.

In order to illustrate this concept, we consider the case of a building management system (depicted in Figure 5). In this case there are CoAP servers located inside buildings and each server is attached to a NAP. Buildings are numbered with a *building* number, and then subdivided into *wings* and *floors*, hence these are the possible group attributes and they are hierarchically organized as shown in the left part of Figure 7. A CoAP client may send a request to a group of CoAP servers; the group name is created by assigning “values” to (some of) the specified attributes, e.g., by setting building=building6, and wing=west, and floor=floor3 the group name *building6.west.floor3* is constructed. POINT NAPs are configured with values for the specified attributes, then by using these values they construct all possible group names e.g., a NAP located in building6, west, 3rd floor, creates the names *building6*, *building6.west*, *building6.floor3*, and *building6.west.floor3*. Then, each NAP subscribes to the ICN content identifier that corresponds to each name. A CoAP request for a group (e.g., *coap://building6.floor2/temperature)*. The CoAP request is encapsulated into a POINT content item and it is advertised in the ICN network using as an identifier the FQDN of the CoAP server as specified in the request’s URL (i.e., in our example ‘building6.floor2’); all NAPs that have subscribed to this identifier will receive that item, will decapsulate the CoAP request and will forward it to the corresponding CoAP servers. Following a similar approach CoAP responses are encapsulated into a POINT content item and are forwarded to the appropriate NAPs and eventually to the interested CoAP clients.

### 5.4. Delayed Response Grouping

In many cases, CoAP clients ask for information that is not yet available, leading to a delayed response. Indeed, the CoAP protocol specifically caters to this pattern by supporting separate acknowledgments for the request and the response. When multiple requests are made by different clients, the server needs to store the pending requests, and when the information becomes available, it needs to send it as a unicast to all the clients. However, the CoAP Handler implementation supports request aggregation transparently to the CoAP server, thus delegating the state management of pending responses to the proxy. In addition, responses to multiple clients are sent in multicast mode over the ICN network, again transparently to the CoAP clients.

### 5.5. CoAP Observe

The details of supporting CoAP observe extension in the NAP of the POINT architecture is presented in [11]. In brief, CoAP requests for the same resource are aggregated in the NAP, hence only a single CoAP client is visible to the CoAP server. In addition, when the value of a resource changes, the update notifications are transmitted using ICN multicast, thus safeguarding network resources. At both ends, these optimizations (e.g., less traffic in the ICN network, less traffic to/from the CoAP server) are transparent to the CoAP clients and server.

**Figure 8:**
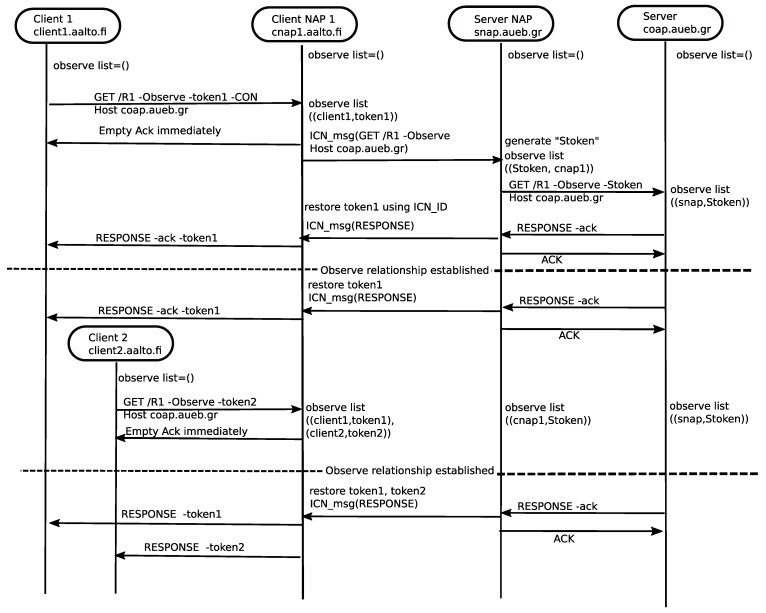
Message Sequence Diagram of CoAP observer.

To illustrate the concept, we present an example of *message sequence diagram* in Figure 8 for two clients and a server and discuss the benefits of ICN underlay to CoAP. First, client1 issues an *observe* request to a CoAP server. cNAP1 receives the request and checks its observe list. If no match is found, it creates an entry for this request in the observe list. cNAP forwards it to sNAP. sNAP receives the request and checks its observe list to find a match. If no match is found, it creates an entry in the observe list. Then, sNAP forwards it to the CoAP server. If the observe relationship is possible, the CoAP server replies with an ACK which eventually creates an observe relationship between CoAP client and CoAP server through NAPs. After some time, another CoAP client (client 2) issues observe request to the same CoAP server. cNAP receives it and finds a match for this request and inserts a new entry in the observe list. Eventually, cNAP does not forward the request to sNAP. cNAP creates an observe relationship with the client 2 on behalf of the CoAP server. Afterwards, cNAP1 forwards all update notification received from the CoAP server to the client 1 and client 2. Similarly, if multiple cNAPs forwards observe requests to the same CoAP server through the same sNAP, sNAP maintains states for those cNAPs. When sNAP receives an update notification from the CoAP server, it forwards the response to all cNAPs using ICN multicast. As a result, ICN can benefit to CoAP observe by shifting both overhead and complexity from the CoAP endpoints to the ICN network.

### 5.6. Limitations

The CoAP protocol runs on top of the UDP. There may be exceptional situations where the client and the server become out of sync due to the latency between the change of the resource state and update notification. In addition, CoAP messages with update notification may get lost. Furthermore, the CoAP server may stop sending update notifications, assuming that the client is no longer interested in the resource subscription. To overcome these exceptional situations, an efficient consistency model is required.

## 6. Related Work

During the last decade, many custom proprietary solutions have been developed and deployed in a multitude of the IoT applications. The interoperability of these solutions and integration of several enabling technologies for the evolution of IoT is a great challenge. It is necessary to have a globally unified IoT platform which enables smart devices or things to connect to the Internet, enabling new forms of communication between humans and things, and between things. The unified IoT platform allows their information and services to be accessible to the standard communication protocols, and sharing information across the physical boundaries of the enterprise. In this context, several research groups in the Internet Engineering Task Force (IETF) are involved in the activity of developing IP-based IoT solutions, such as Low power Wireless Personal Area Networks (6LoWPAN), Routing Over Low power and Lossy networks (ROLL), CoAP [8]. The IETF community related to the IoT technologies has chosen IPv6 to enable wireless communication due to the key features of IPv6, such as extensibility, universality and stability. The 6LoWPAN working group has focused on supporting IPv6 to the MAC layer and the physical layer of IEEE 802.15.4. The RoLL working group focuses on the IPv6 routing protocol for lossy and low power networks (LLN). The CoRE working group has developed CoAP to extend the HTTP web services for the LLN. Recent efforts [15,16] have been performed on proxy based CoAP observe in Wireless Sensor Network (WSNs). However, such IP-based IoT solutions could inherit some of the limitations of IP when dealing with intermittent connectivity and challenged networks [8]. Furthermore, unique identification of objects and the representation and storing of exchanged information in IoT is the most challenging issue.

IoT is increasingly relying on data and information rather than on end-to-end communications, which could ultimately lead to the adoption of ICN architecture and principles. Unlike host-centric IP, every piece of information in ICN is identified by a unique, persistent, location-independent name, which is used by an application for accessing information. In addition, ICN provides security to content itself rather than to a communication channel and enables in-network caching. Such features of ICN provide a great promise to the IoT applications. In recent years, several ICN networking architectures have been proposed for Futrue Internet (e.g., CCN [17], DONA [18], PURSUIT/PSIRP [19], POINT [4], NetInf [20]) with aspiration to efficiently distribute and retrieve content. Various research efforts explore how these architectures can be used in the context of the IoT. At the same time, IPv6 over Low power Wireless Personal Area Networks (6LoWPAN) [21] is a suggested standard to connect Wireless Sensor Networks (WSNs) to the conventional Internet. Considering the IoT from ICN perspective, ICN research group (ICNRG) has involved in the activity of defining requirements and challenges of IoT over the existing ICN proposals [22,23]. Several research efforts [24,25,26,27,28,29,30,31] present the ICN framework in different IoT scenarios. In [24], the authors highlight the key challenges of IoT and provide a design of high level NDN architecture that can meet IoT challenges. In [25], the authors propose the Content-centric Networking (CCN) communication layer on top of MAC layer to transmit packets. Similarly, an overlay of ICN architecture based on CCN on top of ETSI machine-2-machine (M2M) architecture is presented in [26]. In [27], authors propose a service platform based on CCN for Smart Cities that can integrate the available relevant wireless technologies to provide ubiquitous services, optimize the usage of communication resources through distributed caching and provide security by exploiting the security feature of CCN architecture. In [28], the authors provide an experimental comparison of CCN with the traditional IP based IoT standards 6LoWPAN/RPL/UDP in terms of energy consumption and memory footprint. This experiment used a compact version of CCNx [32], referred as CCN-Lite [33], in RIOT OS [34]. The authors of [29] propose a push mechanism for CCN to optimize the traffic in sensor networks, whereas authors of [35] propose a content-centric internetworking scheme for resource-constrained network devices based on task mapping where the network activities (e.g., storing, publishing, and retrieving content) of the constrained devices are transferred to the core CCN network. The work in [30] has designed and implemented a lightweight CCN protocol targeted for Wireless Sensor Network as an alternative to IP protocol for sensor network. All these efforts require modifications to IoT endpoints. In contrast, the POINT architecture provides a CoAP Handler that maps CoAP protocol onto appropriate named objects within the ICN core.

Recent efforts [15,16] have been performed on proxy based CoAP observe in Wireless Sensor Network (WSNs). Alessandro et al. [15] includes WebSocket protocol in the design of the CoAP proxy for HTTP based web applications. The work in [16] considers dynamic aggregation/scheduling of multiple observe requests at CoAP proxies. These efforts are complementary to our work, which utilizes ICN to further enhance the efficiency gains of the CoAP observe.

However, ICN-based approaches in IoT is still in early stage. The deployment of clean-slate ICN in IoT is a great challenge. The clean-slate ICN approach in IoT requires not only the re-design of networking protocols but also the modification of legacy Internet applications. Such radical changes to every network layer are an overwhelming barrier to the adoption of ICN. This paper focuses on an alternative approach which can be deploying ICN solutions as an overlay over the existing IP infrastructure so that ICN features can be exploited to improve the performance of the existing IP protocols in IoT. To achieve this, the paper emphasizes the design, implementation and evaluation of CoAP Handler which provides transparent CoAP services to IoT devices through an ICN core network. The is implemented by the Network Attachment Points (NAPs) that performs translations between the existing IP-based CoAP protocol and appropriately named objects within the ICN core on both edges of the core. In addition, we demonstrate the potential of how CoAP traffic over an ICN network can unleash the full potential of CoAP, shifting both overhead and complexity from the (constrained) endpoints to the ICN network.

## 7. Conclusions and Future Work

In this paper, we present the design and implementation details of the CoAP Handler for the POINT architecture that enable CoAP and CoAP extensions to IoT devices through ICN operator network. In addition, we demonstrate how ICN can benefit CoAP and its extensions, shifting both overhead and complexity from the CoAP endpoints to the ICN network. In addition, we demonstrate how CoAP traffic over an ICN network can unleash the full potential of CoAP, shifting both overhead and complexity from the (constrained) endpoints to the ICN network.

In the case of CoAP observe, our approach aggregates the requests for the same resource and reduces the state management of the CoAP server. Furthermore, our approach also reduces the communication overhead. In the case of CoAP group communication, CoAP server does not need to implement IP multicast. In addition, no modification to DNS is required. Therefore, the resource-constrained IoT device does not need to allocate more resources for a DNS client implementation and IP multicast. Our next steps will be the development of an efficient consistency model in the case of the exceptional situation where the client and the server become out of sync due to the latency between the change of the resource state and update notification.
